# Enhanced Performance of Nanoporous Titanium Dioxide Solar Cells Using Cadmium Sulfide and Poly(3-hexylthiophene) Co-Sensitizers

**DOI:** 10.3390/polym9100467

**Published:** 2017-09-22

**Authors:** Murugathas Thanihaichelvan, Minidu Manoranjana Punya Sri Kodikara, Punniyamoorthy Ravirajan, Dhayalan Velauthapillai

**Affiliations:** 1 Department of Physics, Faculty of Science, University of Jaffna, Jaffna 40000, Sri Lanka; thanihai@jfn.ac.lk (M.T.); mmps19910618@gmail.com (M.M.P.S.K.); 2 Faculty of Engineering, Campus Bergen, Western Norway University of Applied Sciences, P.O. Box 7030, 5020 Bergen, Norway; Dhayalan.Velauthapillai@hvl.no

**Keywords:** hybrid solar cell, titanium dioxide, P3HT, Cadmium Sulfide, chemical bath deposition

## Abstract

This work reports the effect of co-sensitization of nanoporous titanium dioxide using Cadmium Sulfide (CdS) and poly(3-hexylthiophene) (P3HT) on the performance of hybrid solar cells. CdS nanolayer with different thicknesses was grown on Titanium Dioxide (TiO_2_) nanoparticles by chemical bath deposition technique with varying deposition times. Both atomic force microscopy (AFM) and UV–Vis–NIR spectroscopy measurements of TiO_2_ electrode sensitized with and without CdS layer confirm that the existence of CdS layer on TiO_2_ nanoparticles. AFM images of CdS-coated TiO_2_ nanoparticles show that the surface roughness of the TiO_2_ nanoparticle samples decreases with increasing CdS deposition times. Current density–voltage and external quantum efficiency (EQE) measurements were carried out for corresponding solar cells. Both short circuit current density (*J*_SC_) and fill factor were optimized at the CdS deposition time of 12 min. On the other hand, a steady and continuous increment in the open circuit voltage (*V*_OC_) was observed with increasing CdS deposition time and increased up to 0.81 V when the deposition time was 24 min. This may be attributed to the increased gradual separation of P3HT and TiO_2_ phases and their isolation at the interfaces. The higher *V*_OC_ of 0.81 V was due to the higher built-in voltage at the CdS–P3HT interface when compared to that at the TiO_2_–P3HT interface. Optimized nanoporous TiO_2_ solar cells with CdS and P3HT co-sensitizers showed external quantum efficiency (EQE) of over 40% and 80% at the wavelengths corresponding to strong absorption of the polymer and CdS, respectively. The cells showed an overall average efficiency of over 2.4% under the illumination of 70 mW/cm^2^ at AM 1.5 condition.

## 1. Introduction

Hybrid metal oxide/polymer nanocomposites have been under intensive study for potential application in low-cost, durable, and large area solar cells for more than a decade [1,2,3,4]. Hybrid solar cells employ an organic polymer material as an absorber material and a metal oxide electrode as an electron acceptor which improves the chemical stability and durability of these types of cells [5]. Titanium dioxide (TiO_2_) is one of the heavily studied metal oxide electrodes in hybrid, dye, and quantum dot sensitized solar cells due to its wide and direct band gap, environmental friendliness, and transparency in the visible spectra [6,7,8]. Poly(3-hexylthiophene) (P3HT) is an absorber material with an optical band gap of 1.9 eV with absorption spectra peaks at the middle of visible spectra [9]. However, the power conversion efficiencies (PCEs) of TiO_2_–P3HT solar cells are limited by low band-gap of polymers, poor infiltration of polymer into highly structured nanoporous metal oxide, poor matching of polymer absorption spectra with the solar spectrum, high rate of recombination in irregular metal oxide network, and low open-circuit voltages [10,11,12]. The active layer thickness to absorb the light in the cell structure is also limited by lower exciton diffusion lengths and carrier mobility in polymers. Several recent studies were focused on handling these issues to fabricate highly efficient hybrid solar cells. It has been reported that the utilization of oriented nanostructures [13,14], multilayers, and tandem structures [15,16] helped to improve the power conversion efficiencies of hybrid solar cells. Recent studies reveal that photocurrent in these types of solar cells is limited primarily by the photo-generation rate and hence the quality of interface rather mobility of the polymer [17].

The metal oxide–polymer interface is the prime factor that influences carrier separation and recombination in hybrid solar cells. Application of external bias voltage with UV illumination [18], modifying the metal oxide–polymer interface with insulating layers such as alumina [19], self-assembled monolayers [20], other metal oxides [21], and replacing highly structured metal oxides with vertically oriented nanorods [22] are a few strategies adopted to enhance the performance of metal oxide/polymer solar cells. It has also been reported that alumina coating on TiO_2_ nanoparticles has improved the efficiency of TiO_2_/polymer devices by a factor of two. This is attributed to longer carrier lifetime up to 0.5 ms found in alumina incorporated TiO_2_/polymer device as confirmed by photovoltaic transient measurement of alumina coated device and its corresponding control device [19].

More recently, it has been reported [20] that self-assembled monolayers (SAMs) of benzoic acid-based molecules can be used to shift the Fermi level of TiO_2_ and significantly improve short-circuit current density, which is in accordance with the expectation from the driving force for charge separation of titanium dioxide–polymer interface in hybrid TiO_2_–P3HT solar cells. It has been further shown that the SAM layer has dual functions, which are to shift the position of the conduction band of the porous TiO_2_ relative to the polymer HOMO level so as to influence interfacial charge separation and to act as a barrier layer, insulating back electron transfer from the TiO_2_ to the polymer [20]. A few absorbing materials have also been tested as interface modifiers to match the solar spectrum. Most of the materials control the recombination kinetics, while other materials contribute to charge carrier generation [20,23]. It has been reported that the use of CdS nanolayer at the interface of TiO_2_–P3HT extends the spectral response and controls the recombination kinetics simultaneously. The complementing nature of absorption spectra of CdS and P3HT makes them an ideal combination for efficient spectral harvesting. Furthermore, thin CdS layer at the TiO_2_/P3HT interface is found to increase the carrier lifetime to 0.8 ms, which is more than an order of magnitude greater than that in TiO_2_/P3HT devices [24]. Even though the CdS layer plays a dual role in the hybrid solar cells, the thickness of the CdS layer at the interface also has a significant effect due to its defect in nature and strong UV absorption property. Additionally, the addition of a CdS layer at the nanoporous TiO_2_ interface can be achieved at the expense of polymer intake of the TiO_2_ film due to pore filling. This particular work focuses primarily on optimizing the performance of CdS-coated nanoporous TiO_2_/P3HT solar cells to study the role of the CdS layer in photocurrent generation by tailoring the fabrication conditions.

## 2. Materials and Methods

For solar cell fabrication, indium tin oxide (ITO)-coated borosilicate glass substrates (12 mm × 12 mm, ~15 Ω/cm^2^) were cleaned ultrasonically with acetone, isopropanol, and deionized (DI) water. A dense layer of TiO_2_ was then covered on cleaned substrates by spray pyrolysis. The precursor solution for spray pyrolysis was prepared by mixing 1 g of 2,4-pentanedione and 1.42 g of titanium isopropoxide and stirred for completion of the reaction for 15 min. The mixture was then diluted by ten times using absolute ethanol, and 1 mL of this diluted precursor solution was sprayed on dense TiO_2_-covered substrates at 450 °C on a hotplate. The porous TiO_2_ nanocrystalline film of thickness about 600 nm was deposited onto the dense layer by spin coating TiO_2_ paste (DSL 18NRT, Dyesol, NSW, Australia)/tetrahydrofuran solution of concentration 180 mg/mL at 1250 rpm for 30 s. The films were then sintered at 450 °C for 1 h.

**Figure 1:**
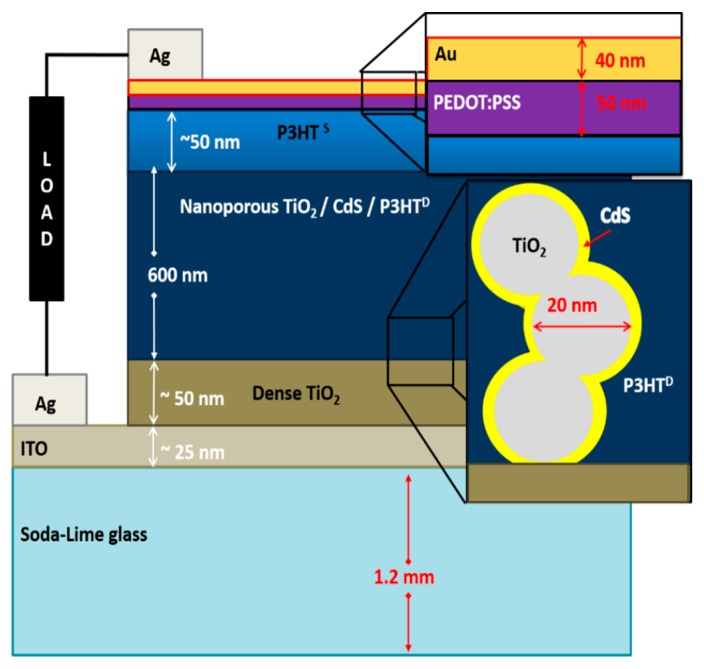
Schematic of the completed device structure with a load (not drawn to scale).

**Figure A1:**
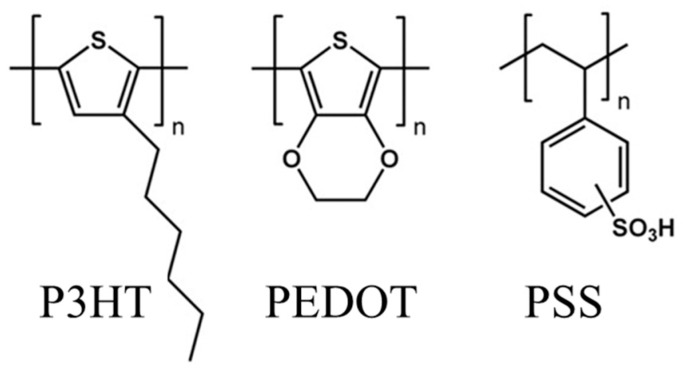
The chemical structures of P3HT and PEDOT:PSS.

The CdS layer was grown onto the nanoporous TiO_2_ layer by chemical bath deposition as described in Refs. [24,25]. Aqueous solutions of 0.033 M cadmium chloride (CdCl_2_), 0.066 M thiourea ((NH_2_)_2_CN), 1 M ammonium chloride (NH_4_Cl), and 1 M ammonium hydroxide (NH_4_OH) were used as precursors. The reaction bath was filled with 250 mL of deionized water, heated to 80 °C, and stirred at a constant rate of 240 rpm using a magnetic stirrer. The substrates were kept vertically so that the TiO_2_ deposited surface faced the center of the reaction bath. The prepared solutions of 8 mL NH_4_OH, 4 mL CdCl_2_, and 2 mL NH_4_Cl were added in an interval of 1 min and the temperature of the bath was raised up to 85 °C. The thiourea solution was then titrated by 1 mL doses for four times in an interval of 1 min. The system was kept at a constant temperature of 85 °C until the samples were removed. CdS layers with different layer thicknesses were coated on the TiO_2_ electrode by depositing CdS for 8, 12, 16, and 24 min after the last titration of thiourea solution. The samples were washed in DI water, flushed with dry nitrogen, and baked at 320 °C for the removal of excess water and chemical residues in the film. The bare and CdS-grown films were then immersed in 1 mg/mL P3HT solution in 1,2-dichlorobenzene (DCB) for 2 h at 120 °C and a layer of P3HT was then spin-coated onto the immersed electrodes using 25 mg/mL P3HT solution in DCB at 4000 rpm for 30 s. Poly(3,4-ethylenedioxythiophene) polystyrene sulfonate (PEDOT:PSS) layer was deposited over the P3HT layer by spin-coating filtered PEDOT:PSS solution at 4000 rpm for 30 s. A 40 nm gold layer was deposited by thermal evaporation under high vacuum (2 × 10^−6^) to form the top contact using an Edwards E306 thermal evaporator. Figure 1 shows the schematic of the fabricated cell structure (See Figure A1 for the chemical structures of P3HT and PEDOT:PSS).

The optical absorption spectra of the CdS-deposited nanoporous TiO_2_ electrodes and CdS–TiO_2_ electrodes after dipping into P3HT solution were obtained by using UV–Vis spectrometer (JENWAY-6800, Staffordshire, UK). Atomic force microscopy (AFM) (Park, Suwon, Korea) images were taken using dynamic force tapping mode, and surface roughness of the films were measured from the images scanned over the area of 10 µm × 10 µm. Current–voltage (I–V) measurements were done on fabricated solar cells using a computer interfaced source-measure unit (Keithly 2400, Cleveland, OH, USA) and a solar simulator (SCIENCETECH, London, ON, Canada). External quantum efficiency (EQE) measurements were carried out with a calibrated silicon photodiode (Newport, Irvine, CA, USA) and a monochromator (Newport, Irvine, CA, USA). To ensure the reproducibility, 12 devices in each reposition time were fabricated, and the average values are reported in the discussion.

## 3. Results and Discussion

**Figure 2:**
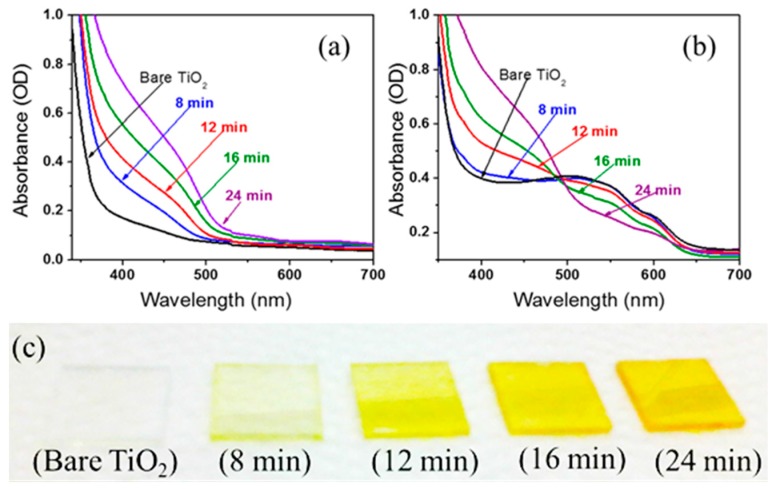
(**a**) UV–Vis–NIR spectra of bare TiO_2_ film and CdS-deposited nanoporous TiO_2_ films with deposition times of 8, 12, 16, and 24 min prior to deposition of P3HT; (**b**) After dipping in P3HT solution for 2 h; and (**c**) Photograph of nanoporous TiO_2_ films coated with CdS layer with different deposition times prior to deposition of P3HT.

Figure 2a shows the UV–Vis–NIR (ultraviolet–visible–near-infrared) spectra of bare TiO_2_ film and CdS-deposited nanoporous TiO_2_ films with deposition times of 8, 12, 16, and 24 min before deposition of P3HT. The strong absorption edge of 520 nm ensures the presence of direct band gap CdS in the films [24,25]. The absorption peak of CdS in the UV region increases with the increment of CdS layer in the nanoporous TiO_2_ electrode with increased deposition time, as reported in [26,27]. Figure 2b illustrates the UV–Vis–NIR spectra of bare TiO_2_ film and CdS-deposited nanoporous TiO_2_ films with deposition times of 8, 12, 16, and 24 min after dipping in P3HT solution for 2 h. The broad peak at 510 nm and the shoulder at around 570 nm regardless of the deposition time of CdS confirm the presence of P3HT [28]. The reduction in optical absorbance in these films with CdS deposition can be attributed to the reduction of voids in the nanoporous electrodes, which in turn reduce the polymer adsorption. The photograph of samples fabricated with different CdS deposition times is depicted in Figure 2c. The aggregated amount of CdS in nanoporous TiO_2_ films can be observed from the color change in the films.

**Figure 3:**
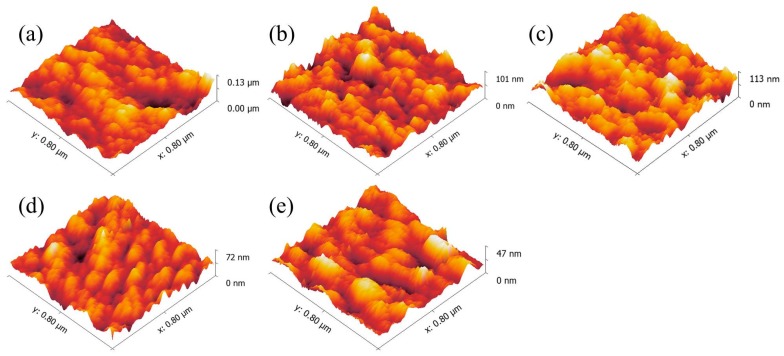
Atomic force microscopy (AFM) images of (**a**) bare and CdS layer-coated TiO_2_ nanoporous films with CdS deposition times of (**b**) 8, (**c**) 12, (**d**) 16, and (**e**) 24 min.

Figure 3a–e show the surface morphology of spin-coated nanoporous TiO_2_ layer and CdS-deposited nanoporous TiO_2_ layers. The surface roughnesses of the samples were found to be 119, 105, 92, 70, and 60 nm for bare and CdS layer-coated TiO_2_ layers for 8, 12, 16, and 24 min, respectively. The decreasing trend in roughness with increasing deposition time can be explained by the addition of fine CdS crystals on the rough TiO_2_ surface that resulted in pore filling. The surface of the CdS-coated TiO_2_ layer for 8 and 12 min shows the coarse structure which ensures the availability of partially filled pores. AFM images of the CdS-coated TiO_2_ layer for 16 and 24 min clearly show the formation of clusters of particle agglomerates over the TiO_2_ layer and filled pores which are reflected in the polymer intake. The huge particle agglomerations and smooth surfaces ensure that the CdS covers the surface of the nanoporous TiO_2_ layer.

**Figure 4:**
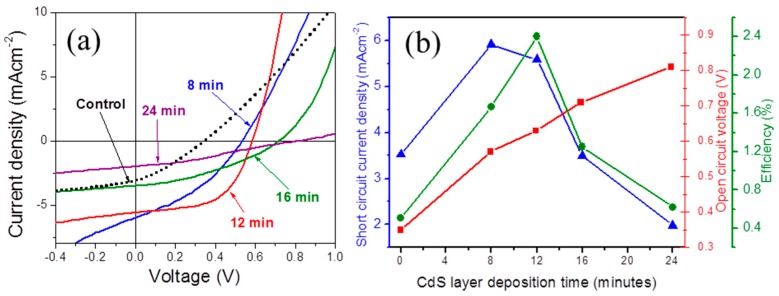
(**a**) J–V measurements of the TiO_2_/CdS/P3HT solar cells with different CdS layer deposition times; and (**b**) The variation of *J*_SC_ (Triangle), *V*_OC_ (Square), and power conversion efficiency (PCE, circle) of TiO_2_/CdS/P3HT solar cells with CdS deposition time.

J–V characteristics of “CdS deposition time varied TiO_2_/CdS/P3HT” solar cells and the respective variation of average values of *J*_SC_, *V*_OC_, and efficiency are depicted in Figure 4a,b, respectively. Figure 4b clearly shows the steady increment in the *V*_OC_ value with increased CdS deposition time, while *J*_SC_ exhibits an increasing trend with CdS deposition time optimized at 8 min, and thereafter a decreasing trend with increasing deposition time. The increasing trend in the *V*_OC_ with CdS deposition time can be explained by the insertion of CdS layer and the increase of CdS layer thickness at the TiO_2_–P3HT interface. The *V*_OC_ of 0.81 V was achieved when the TiO_2_–P3HT interface was almost isolated due to the CdS layer, as shown in the AFM images. This is due to the increased separation between the donor’s HOMO and acceptor’s conduction band edge of acceptor from 0.7 to 1.1 eV, which is consistent with the higher *V*_OC_ found in TiO_2_/CdS/P3HT devices [24]. This signifies the greater involvement of CdS in carrier generation in the devices. The increased *V*_OC_ can also be attributed to reduced interfacial recombination. When the deposition time reached 24 min, the average *J*_SC_ dropped to 1.9 mA/cm^2^, which is lower than that offered by the control device. A similar trend to that exhibited by *J*_SC_ was observed for fill factor of the cells, which evidenced that the quality of the TiO_2_/polymer interface was optimized at the CdS deposition time of 12 min. The maximum average PCE of 2.4% was obtained with a CdS layer deposition time of 12 min, *J*_SC_ of 5.6 mA/cm^2^, *V*_OC_ of 0.63 V, and fill factor of 0.49 under the illumination of 70 mW/cm^2^ at AM 1.5 conditions (see Table A1 for the estimated short circuit current densities of devices from the spectral parameters of 100 mW/cm^2^ at AM 1.5 and external quantum efficiency spectra of the corresponding devices).

**Figure 5:**
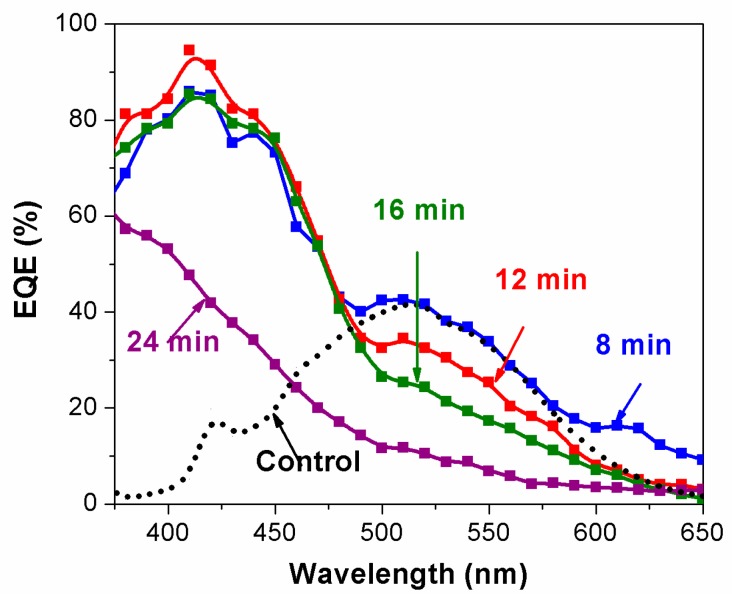
External quantum efficiency (EQE) spectra of solar cells fabricated with TiO_2_ films with different CdS deposition times.

**Figure A2:**
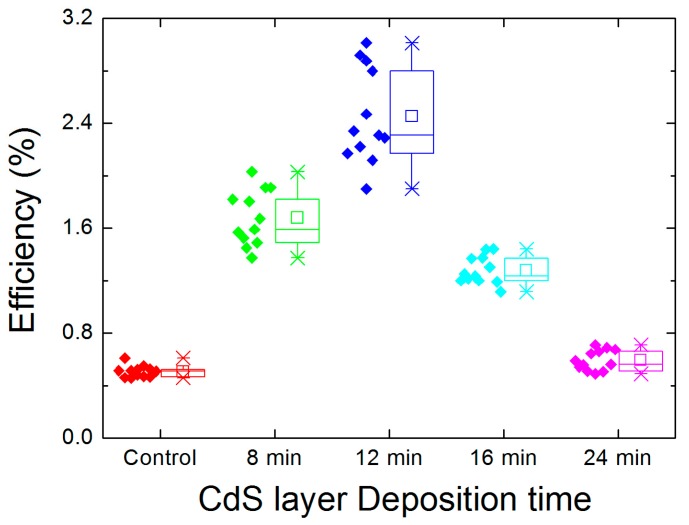
The power conversion efficiency distribution of 12 fabricated devices in each CdS deposition time. Control represents the devices without CdS layer. The values reported in the main text are average values unless stated.

The spectral response of the devices was studied by using the corresponding EQE spectra. Figure 5 shows the EQE spectra of solar cells made with TiO_2_ films with different CdS deposition times. The cell without a CdS layer shows the EQE spectra of TiO_2_–P3HT solar cells with two peaks at the wavelengths corresponds to strong absorption of P3HT and TiO_2_. The device fabricated with 8 min CdS deposition time shows a new peak in EQE spectra at the absorption peak of CdS, whereas the changes in EQE at the absorption of P3HT are negligibly small when compared to that of TiO_2_–P3HT solar cells. This confirms the stronger involvement of CdS layer in photo-current generation. EQE values of the cells with 8 min CdS layer deposition time showed over 40% and 80% at the wavelength corresponding to strong absorption of P3HT and CdS, respectively. This indicates the role of polymer in photo-current generation with reduced polymer absorption, which can be attributed to the active involvement of CdS–P3HT heterojunction in free carrier generation [29,30,31]. Although EQE spectra resembled both absorption of CdS and P3HT, higher EQE was found at the peak absorption of CdS than EQE at the peak absorption of P3HT, suggesting efficient photovoltaic action was due to CdS interlayer, which is consistent with the literature [26]. EQE spectra showed a steady decrement in EQE values at the absorption peak of P3HT (~520 nm) with increasing deposition time up to 16 min in accordance with the corresponding absorption spectra of CdS and decreased polymer intake due to CdS-filled pores. There were no significant changes in the EQE at the absorption of CdS during the same interval, and this can be attributed to the saturation of CdS layer. However, EQE values at the peak absorption of CdS:P3HT composite were reduced dramatically when the deposition time was increased to 24 min. This may be due to the filtering effect of thicker CdS layer that reduces the effective charge separation yield at the interface. Higher *V*_OC_ of the solar cells with 24 min CdS layer deposition time confirms the separation of TiO_2_ and P3HT, which is the major photocurrent generating interface in this structure. The EQE in the P3HT absorption region almost vanishes in these devices, and it can also be an outcome of lowered interfacial area due to pore filling. An optimized TiO_2_/CdS/P3HT device with 12 min CdS deposition time shows the overall average cell efficiency of over 2.4% with a champion cell efficiency of over 3.2% (see Figure A2 for the efficiency distribution of 12 devices in each deposition time) under the illumination of 70 mW/cm^2^ at AM 1.5 conditions. This is considerably higher than that of previously reported TiO_2_/CdS/P3HT solid state cells with or without additional interface modifiers or dopants [11,29,32,33] and TiO_2_/CdS/P3HT solar cells with liquid electrolytes [31,34].

## 4. Conclusions

The performance of hybrid TiO_2_/P3HT solar cells can be improved by systematically controlling the thickness of CdS co-sensitizing interlayer. The role of the CdS interlayer is attributed to extended spectral response, smooth charge transfer, suppressed interfacial charge recombination, increased built-in voltage, and number of dissociation sites available for charge carrier generation. Optimized TiO_2_ solar cells with CdS and P3HT co-sensitizer showed EQE of over 40% and 80% at the wavelengths corresponding to strong absorption of the polymer and CdS, respectively. The cell showed an overall average efficiency of over 2.4% under the illumination of 70 mW/cm^2^ at AM 1.5 condition.

## Appendix A

**Table A1:** The measured *J*_SC_ values at simulated irradiation of 70 mW/cm^2^ with AM 1.5 filter and the calculated *J*_SC_ using spectral properties of 100 mW/cm^2^ irradiation at AM 1.5 conditions from the EQE spectra of the same cells.

CdS deposition time	Measured *J*_SC_ (mA/cm^2^)	Calculated *J*_SC_ from EQE spectra (mA/cm^2^)
0 min (Control)	3.08	3.48
8 min	5.97	7.24
12 min	5.6	6.86
16 min	3.5	4.17
24 min	1.98	2.76
